# Prostate-specific membrane antigen (PSMA) as a potential target for molecular imaging and treatment in bone and soft tissue sarcomas

**DOI:** 10.1259/bjr.20220886

**Published:** 2023-03-03

**Authors:** Fleur Kleiburg, Linda Heijmen, Hans Gelderblom, Szymon M Kielbasa, Judith VMG Bovée, Lioe-Fee De Geus-Oei

**Affiliations:** 1 Biomedical Photonic Imaging Group, University of Twente, Enschede, The Netherlands; 2 Department of Radiology, section of Nuclear Medicine, Leiden University Medical Center, Leiden, The Netherlands; 3 Department of Medical Oncology, Leiden University Medical Center, Leiden, The Netherlands; 4 Department of Biomedical Data Sciences, Leiden University Medical Center, Leiden, The Netherlands; 5 Department of Pathology, Leiden University Medical Center, Leiden, The Netherlands; 6 Department of Radiation Science and Technology, Technical University of Delft, Delft, The Netherlands

## Abstract

Bone and soft tissue sarcomas are a group of rare malignant tumours with major histological and anatomical varieties. In a metastatic setting, sarcomas have a poor prognosis due to limited response rates to chemotherapy. Radioligand therapy targeting prostate-specific membrane antigen (PSMA) may offer a new perspective. PSMA is a type II transmembrane glycoprotein which is present in all prostatic tissue and overexpressed in prostate cancer. Despite the name, PSMA is not prostate-specific. PSMA expression is also found in a multitude of non-prostatic diseases including a subgroup of sarcomas, mostly in its neovascular endothelial cells. On PET/CT imaging, multiple sarcomas have also shown intense PSMA-tracer accumulation. PSMA expression and PSMA-tracer uptake seem to be highest in patients with aggressive and advanced sarcomas, who are also in highest need of new therapeutic options. Although these results provide a good rationale for the future use of PSMA-targeted radioligand therapy in a selection of sarcoma patients, more research is needed to gain insight into optimal patient selection methods, PSMA-targeting antibodies and tracers, administered doses of radioligand therapy, and their efficacy and tolerability. In this review, mRNA expression of the FOLH1 gene which encodes PSMA, PSMA immunohistochemistry, PSMA-targeted imaging and PSMA-targeted therapy in sarcomas will be discussed.

## Introduction

Sarcomas are a diverse group of malignant tumours that arise from connective tissues, such as bone, cartilage, fat, muscle and blood vessels. These tumours can occur in many different anatomic locations and currently, over 70 histological subtypes are defined.^1^ Sarcomas can be divided into two broad categories: soft tissue sarcomas and bone sarcomas, with an estimated incidence of 4.7 per 100,000 /year and 0.8 per 100,000 /year, respectively.^2^ Together they represent less than 1% of all new cancer cases.^3,4^ Due to its rarity and its histological and anatomical heterogeneity, diagnosis and optimal tumour management are challenging. Individual treatment plans of sarcoma patients should therefore always be made in multidisciplinary teams of sarcoma reference centres.^5,6^ The golden standard for localized intermediate and high-grade sarcomas is complete surgical resection with wide and microscopically tumour-free (R0) margins. Selected patients, *e.g*. in case of high-risk lesions, can be treated additionally with (neo)adjuvant radiotherapy and/or chemotherapy to improve clinical outcomes. Although complete removal of the tumour is pursued, unfortunately around 30–50% of patients, also depending on tumour characteristics, still develop local recurrences and/or metastases after primary treatment.^7–10^ In addition, at least 14–17% of sarcoma patients have distant metastases at presentation.^11^ Standard of care in patients with metastatic sarcomas consists of cytotoxic chemotherapy, with doxorubicin being the first choice in most sarcoma types.^5,6^ With the administration of doxorubicin, in combination with or without ifosfamide chemotherapy, treatment response rates of approximately 25% are reached in advanced soft tissue sarcoma^12,13^ and even lower in bone sarcomas. Due to these low response rates to chemotherapy, the five-year survival rates in metastatic soft tissue sarcoma and bone sarcoma are 17 and 31%, respectively.^14^ This shows that there is a high need for new effective treatment options that can decrease burden of disease and increase survival, especially in sarcoma patients with advanced disease.

Prostate-specific membrane antigen (PSMA) may offer a new perspective for sarcoma patients. Despite the name, PSMA is not prostate cancer-specific. PSMA, also known as glutamate carboxypeptidase II, is a type II transmembrane glycoprotein which consists of 750 amino acids and is encoded by the FOLH1 (folate hydrolase 1) gene.^15^ In prostatic tissue, PSMA is expressed in the secretory epithelial cells, and PSMA is overexpressed in prostate cancer. Further upregulation of PSMA expression is seen in more advanced prostate cancers and PSMA was shown to be an independent predictor of poor prognosis.^16^ Although the exact mechanisms are unknown, PSMA is associated with the activation of PI3K/AKT and cAMP/PKA pathways, which are involved in cell proliferation.^17,18^ The expression pattern of PSMA has made it a well-established target for molecular imaging in prostate cancer. In the last decade, PSMA-targeting PET/CT scans have found their way into clinical practice for primary staging of high-risk prostate cancer patients and in case of biochemical recurrence after primary treatment.^19^ Furthermore, PSMA targeting ligands have been labelled with therapeutic nuclides such as lutetium-177 or actinium-225, and these radioligand therapies have achieved beneficial effects in advanced prostate cancer patients with acceptable toxicity.^20,21^ Interestingly, PSMA has been found in the tumour-associated neovascular endothelial cells of a wide variety of other tumours besides prostate cancer, such as renal cell carcinoma, glioblastoma, hepatocellular carcinoma, thyroid cancer, lung cancer, breast cancer, and sarcoma.^22^ Since PSMA-targeted radioligand therapy (PSMA-RLT) has demonstrated promising results in patients with prostate cancer, the question arises whether this therapy could also have a beneficial effect in other PSMA-positive tumours, such as sarcomas.

This review aims to give an overview of the available literature about the possible role of PSMA in sarcomas. mRNA expression of the FOLH1 gene, immunohistochemical PSMA expression, PSMA-targeted imaging, PSMA-targeted therapy and the possible future perspectives for sarcomas will be discussed.

## Methods

For the literature search, the following search strategy was used in PubMed:

(“Glutamate carboxypeptidase II”[MeSH] OR “Glutamate carboxypeptidase II”[tiab] OR “PSMA”[tiab] OR “Prostate-specific membrane antigen”[tiab] OR “prostate specific membrane antigen”[tiab]) AND (“sarcoma”[MeSH] OR “sarcoma”[tiab] OR “sarcomas”[tiab])

This resulted in 28 articles, of which 15 were relevant for the topic of this review. The excluded articles were either not about PSMA in sarcomas (*n* = 11), or were reviews that cited already included articles (*n* = 2). The same search strategy was used in Scopus and Web of Science, which did not add other relevant articles. Also, no extra articles were found while reading the included articles and their references. PubMed, Scopus and Web of Science were last checked for new articles on 05-12-2022.

### mRNA expression of the FOLH1 gene in sarcomas

**Figure 1.**
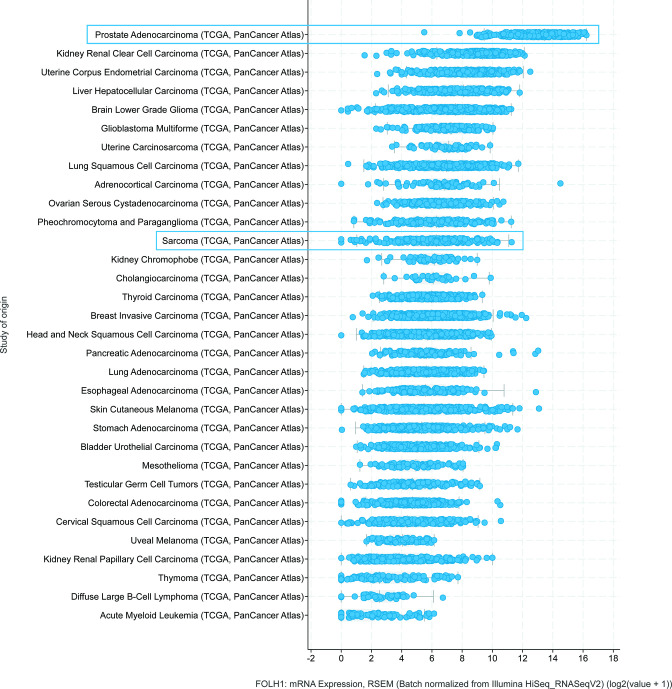
Log transformed FOLH1 mRNA expression levels of the 32 tumour types from the TCGA Pan-Cancer Atlas. Sorted by the median in descending order. This figure was obtained from cBioportal.org.^24,25^

PSMA is encoded by the FOLH1 gene, which is localized on chromosome 11p11-p12 and contains 19 exons.^23^ As part of the large-scale Pan-Cancer analysis project from The Cancer Genome Atlas Research Network, the FOLH1 mRNA expression levels were determined in over 10.000 tumours from 32 different tumour types, together with a wide variety of other genetic and clinical data. Figure 1 shows the results from this analysis, obtained from cBioPortal.^24,25^ The FOLH1 mRNA expression levels were calculated by the logarithmic transformation of FOLH1 abundance estimates using RSEM.^26^ After sortation of FOLH1 mRNA expression levels by median, sarcomas were 12^th^ of the 33 tumour types. There still was a notable difference between the FOLH1 mRNA expression levels in prostate carcinomas and all other carcinomas, which can be seen in Figure 1.

**Figure 2.**
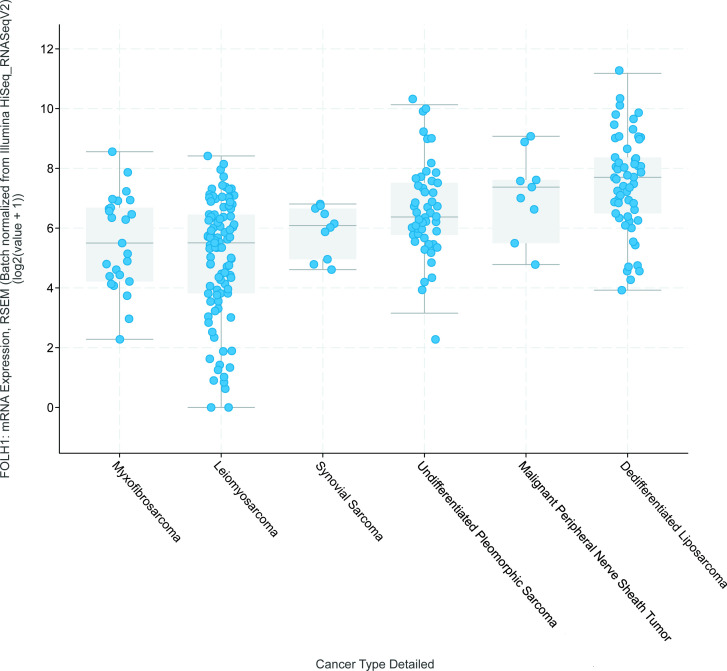
Log transformed FOLH1 mRNA expression levels of myxofibrosarcoma, leiomyosarcoma, synovial sarcoma, undifferentiated pleomorphic sarcoma, malignant peripheral nerve sheath tumour and dedifferentiated liposarcoma. Sorted by the median in ascending order. Of the sarcomas, dedifferentiated liposarcomas had the highest FOLH1 mRNA expression levels. This figure was obtained from cBioportal.org.^24,25^

The 251 sarcomas within the Pan-Cancer Atlas were further divided into different subtypes: leiomyosarcoma (*n* = 99), dedifferentiated liposarcoma (*n* = 58), undifferentiated pleomorphic sarcoma (*n* = 50), myxofibrosarcoma (*n* = 25), synovial sarcoma (*n* = 10) and malignant peripheral nerve sheath tumour (*n* = 9). Thus, this means that only a small subset of soft tissue sarcoma subtypes and no bone sarcomas were represented in this sarcoma database. There was a statistically significant difference in FOLH1 mRNA expression levels between the different sarcoma types (Kruskal-Wallis test, H(5) = 65.587, *p* < 0.001). Dedifferentiated liposarcomas had higher FOLH1 mRNA expression levels (median = 7.7) compared to malignant peripheral nerve sheath tumours (median = 7.4), undifferentiated pleomorphic sarcomas (median = 6.4), synovial sarcoma (median = 6.1), myxofibrosarcoma (median = 5.5) and leiomyosarcoma (median = 5.5). The maximum FOLH1 mRNA expression level was also highest in dedifferentiated liposarcomas, followed by undifferentiated pleomorphic sarcomas (Figure 2).

**Figure 3.**
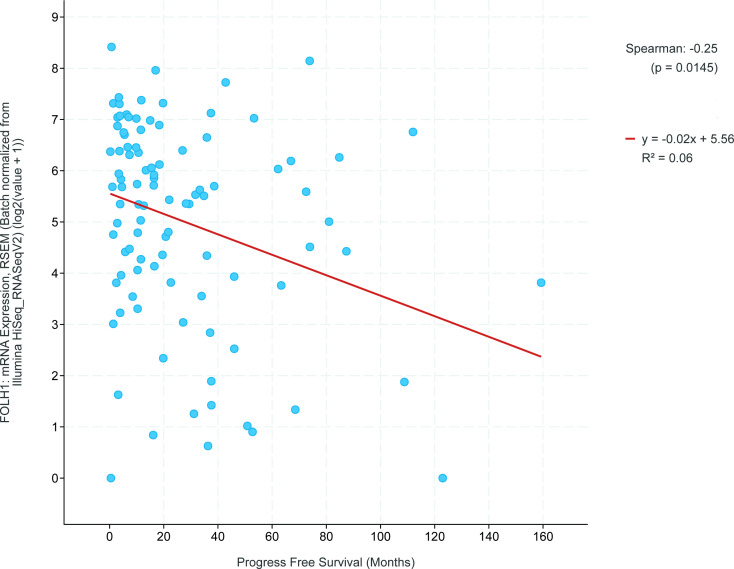
Correlation between log-transformed FOLH1 mRNA expression levels and months of progression-free survival in 100 leiomyosarcomas. Spearman correlation −0.25, *p* = 0.015. R^2^ = 0.06. This figure was obtained from cBioportal.org.^24,25^

The Pan-Cancer Atlas also contained overall survival and progression-free survival data. Within the leiomyosarcoma group, higher FOLH1 mRNA expression levels correlated significantly with shorter progression-free survival (Spearman correlation = −0.25, *p* = 0.015, Figure 3). However, R^2^ was 0.06, so only a small percentage of the variation could be attributed to the FOLH1 mRNA expression levels. No such correlation was seen with overall survival. In the other sarcoma subtypes, no correlation was found between FOLH1 mRNA expression levels and progression-free survival or overall survival.

### PSMA immunohistochemistry in sarcomas

For determining PSMA expression in formalin-fixed paraffin-embedded (FFPE) tissue samples, immunohistochemistry is the designated technique. Up until now, four articles have investigated immunohistochemical PSMA expression in sarcomas.

Heitkötter et al. analysed 779 samples from 25 different soft tissue and bone tumour types,^27^ of which 599 were sarcomas. PSMA expression was found to be positive in 151 of 779 soft tissue and bone tumours (19.4%). Similar to other non-prostate tumours, this PSMA expression was found in the tumour-associated neovasculature and was more frequent in sarcomas compared to soft tissue and bone tumours with benign or intermediate biological potential. Strong PSMA expression, defined as moderate staining (readily apparent at 40x magnification) in >5% of the neovasculature or any strong staining of the neovasculature, was found in 43 of 779 soft tissue and bone tumours (5.5%). Table 1 lists the sarcoma subtypes of which over 20% of analysed tumour samples showed any PSMA expression. The sarcoma subtypes with the highest frequency of PSMA-positivity were pleomorphic rhabdomyosarcoma (60%), synovial sarcoma (56%) and pleomorphic liposarcoma (50%). These were also the sarcoma types with the highest number of tumours with strong PSMA expression (40%, 38 and 20%, respectively). As for bone sarcomas, 106 Ewing sarcomas were investigated, of which six were PSMA-positive and none showed strong PSMA expression. No other bone sarcomas were included.

**Table 1.** Immunohistochemical PSMA expression in different sarcoma subtypes. Different monoclonal antibodies were used.

Authors	Used monoclonal antibody	Sarcoma subtype	Number of tumours with any PSMA expression	Number of tumours with strong PSMA expression ^a^
Heitkötter et al.^27^ ^b^	3E6	Pleomorphic rhabdomyosarcoma	3/5 (60%)	2/5 (40%)
		Synovial sarcoma	9/16 (56%)	6/16 (38%)
		Pleomorphic liposarcoma	5/10 (50%)	2/10 (20%)
		Undifferentiated pleomorphic sarcoma	15/33 (46%)	6/33 (18%)
		Malignant peripheral nerve sheath tumour	7/21 (33%)	4/21 (19%)
		Leiomyosarcoma	21/66 (32%)	7/66 (11%)
		Endometrial stromal sarcoma	1/4 (25%)	0/4 (0%)
		Dedifferentiated liposarcoma	17/75 (23%)	0/75 (0%)
Zeng et al.^28^	Unknown	Osteosarcoma	21/45 (47%)	-
Chang et al.^29^	7E11 PM2J004.5 J591 J415 PEQ226.5	Soft tissue sarcoma (specific subtype unknown)	6/7 (86%) ^c^	-
Coskun et al.^30^	EP192	Malignant peripheral nerve sheath tumour	0/25 (0%)	-

a Strong PSMA expression was defined as either moderate staining (readily apparent at 40x magnification) in>5% of the neovasculature or any strong staining of the neovasculature.

b From this study, the sarcoma subtypes of which over 20% of analysed tumours were PSMA-positive are included in this table. Additionally, the number of tumours with strong PSMA expression is described in these sarcoma subtypes. All PSMA expression was found in the tumour’s neovasculature.

c All five monoclonal antibodies gave the same results.

Zeng et al. investigated PSMA expression in osteosarcomas.^28^ After immunohistochemical analysis, 21 of 45 osteosarcomas (47%) demonstrated PSMA reactivity in the tumour-associated neovasculature, not in the tumour cells. Interestingly, PSMA expression was significantly associated with tumour size (*p* = 0.042), the presence of pulmonary metastasis (*p* < 0.001) and a worse 5 year survival rate (36.6% *vs* 63.2%, *p* < 0.05). These findings support the hypothesis that PSMA expression is associated with worse clinical outcome. PSMA expression was not associated with age, gender and location. In two other studies, sarcomas were studied as part of a larger tissue sample cohort. Chang et al. studied 20 benign and 12 malignant tissue types, including seven soft tissue sarcomas.^29^ Of these, six turned out PSMA-positive. No further histological information on these soft tissue sarcomas is described. Coskun et al. investigated PSMA expression, along with STAT3 and VEGF expression, in 25 malignant peripheral nerve sheath tumours (MPNST).^30^ None of the 25 MPNSTs were PSMA-positive.

It is important to take into account that up until now, a wide variety of monoclonal antibodies (mAbs) have been developed to use for PSMA immunohistochemistry and that in the previously described articles, several different mAbs are used (Table 1) . The 7E11 mAb was the firstly used anti-PSMA antibody, which binds to the intracellular epitope of PSMA. More recently developed mAbs, such as mAb 3E6, used by Heitkötter et al.,^27^ often bind to the extracellular epitope of PSMA. Chang et al.^29^ compared two mAbs that bind to the intracellular PSMA domain (7E11 and PM2J004.5) to three other mAbs that bind to the extracellular PSMA domain (J591, J415 and PEQ226.5). All five anti-PSMA mAbs reacted strongly with the neovasculature of the analysed malignant tumours, including soft tissue sarcomas, so there was no difference found by these authors. Coskun et al.^30^ describe that 0/25 (0%) MPNSTs showed any PSMA expression, while Heitkötter et al.^27^ report that 7/21 (33%) MPNSTs were PSMA-positive, by the use of mAb EP192 and mAb 3E6, respectively. It is difficult to conclude if the mAb may account for this difference, as no comparative studies between these mAbs have been conducted.

When looking specifically at the seven different soft tissue sarcoma subtypes of which FOLH1 mRNA expression levels have been determined in the Pan-Cancer Atlas, it is noticeable that the mean FOLH1 mRNA expression levels do not necessarily correspond to the immunohistochemical PSMA positivity rates from Heitkötter et al. Dedifferentiated liposarcomas had the highest mean FOLH1 mRNA expression, but only 22.67% of the investigated dedifferentiated liposarcomas showed any PSMA expression and none showed strong PSMA expression. On the other hand, synovial sarcomas were only fifth in median FOLH1 mRNA expression but had the highest number of PSMA-positive tumours (56.3%) and tumours with strong PSMA expression (37.5%) of these seven subtypes. Thus, based on these limited data that are available, there does not seem to be a correlation between mRNA expressions of FOLH1 and protein expression of PSMA.

### PSMA-targeted PET/CT imaging in sarcomas

Because there is evidence available about the presence of PSMA in sarcomas, the question arises whether PSMA-tracer uptake can be seen on the PSMA-targeted PET/CT scans that have been developed for prostate cancer. Available literature about PSMA-targeted imaging in sarcomas consists of case reports, as no case series or prospective studies have been published yet. Table 2 describes each of these case reports.

**Table 2.** An overview of all published case reports describing PSMA-tracer accumulation in sarcomas on PET/CT imaging. The SUV_max_ values are included in this table if described in the case report

Authors	Gender	Age	Scan	Diagnosis	Description of PSMA-tracer uptake	Other
Malik et al.^31^	Male	76	^68^Ga-PSMA PET/CT	Well-differentiated liposarcoma in the left sartorius muscle	Mild uptake	
Inanir et al.^32^	Male	77	^68^Ga-PSMA PET/CT	Dedifferentiated liposarcoma, 42 cm (craniocaudally) retroperitoneal mass	Visible uptake in the non-lipomatous component, but no uptake in the lipomatous region	[^18^F]FDG PET/CT matched findings, but with a lower tumour-to-background ratio
Militano et al.^33^	Male	74	^68^Ga-PSMA PET/CT	Pleomorphic liposarcoma of the left quadriceps	SUV_max_ = 13	8.5 × 6×11.2 cm mass
Plouznikoff et al.^34^	Male	67	^68^Ga-PSMA PET/CT	Undifferentiated soft tissue sarcoma in the left internal obturator muscle, most likely radiation-induced	Visible uptake above the background	FNCLCC Grade 2
Mathew et al.^35^	Male	72	^68^Ga-PSMA PET/CT	High-grade pleomorphic sarcoma in the right posterior chest wall	Intense PSMA uptake	
Parihar et al.^36^	Female	19	[^68^Ga]Ga-PSMA-11 PET/CT	Ewing sarcoma in the left iliac bone	SUV_max_ = 7.4	[^18^F]FDG PET/CT: SUV_max_ = 5.1
Sasikumar et al.^37^	Female	60	^68^Ga-PSMA PET/CT	Fibrous dysplasia with areas of osteosarcoma in left side-skull bones	Clear uptake in osteosarcoma, not in fibrous dysplasia	99mTC-MDP: could not differentiate between osteosarcoma and fibrous dysplasia
Can et al.^38^	Male	75	^68^Ga-PSMA PET/CT	High-grade osteosarcoma in the sternum with multiple lung, bone and liver metastases	SUV_max_ = 16.9 (sternum), 10.6 (lung), 23.1 (bone), 35.1 (liver)	[^18^F]FDG PET/CT: SUV_max_ = 14.6 (sternum), 3.1 (lung), 9.5 (bone), 7.9 (liver)
Marafi et al.^39^	Male	59	^18^F-PSMA PET/CT	Angiosarcoma in the scalp with metastases in ribs, iliac bones, femur and right tibia	Increased uptake	
Juptner et al.^40^	Female	50	^68^Ga-PSMA PET/CT	Leiomyosarcoma of the vena cava inferior with metastases in bone, lung, liver and muscle	SUV_max_ = 14.5 (bone), 1.7 (lung), 12.9 (liver), 16.5 (gluteus maximus muscle), 3.7 (vastus lateralis muscle)	Received one application of 6.0 GBq [^177^Lu]Lu-PSMA-617
Digklia et al.^41^	Female	Mid-50s	[^68^Ga]Ga-PSMA-11 PET/CT	Uterine leiomyosarcoma with pulmonary and peritoneal metastases	SUV_max_ = 8.9 (lung)	Received two applications of [^177^Lu]Lu-PSMA-I&T in combination with nivolumab

**Figure 4.**
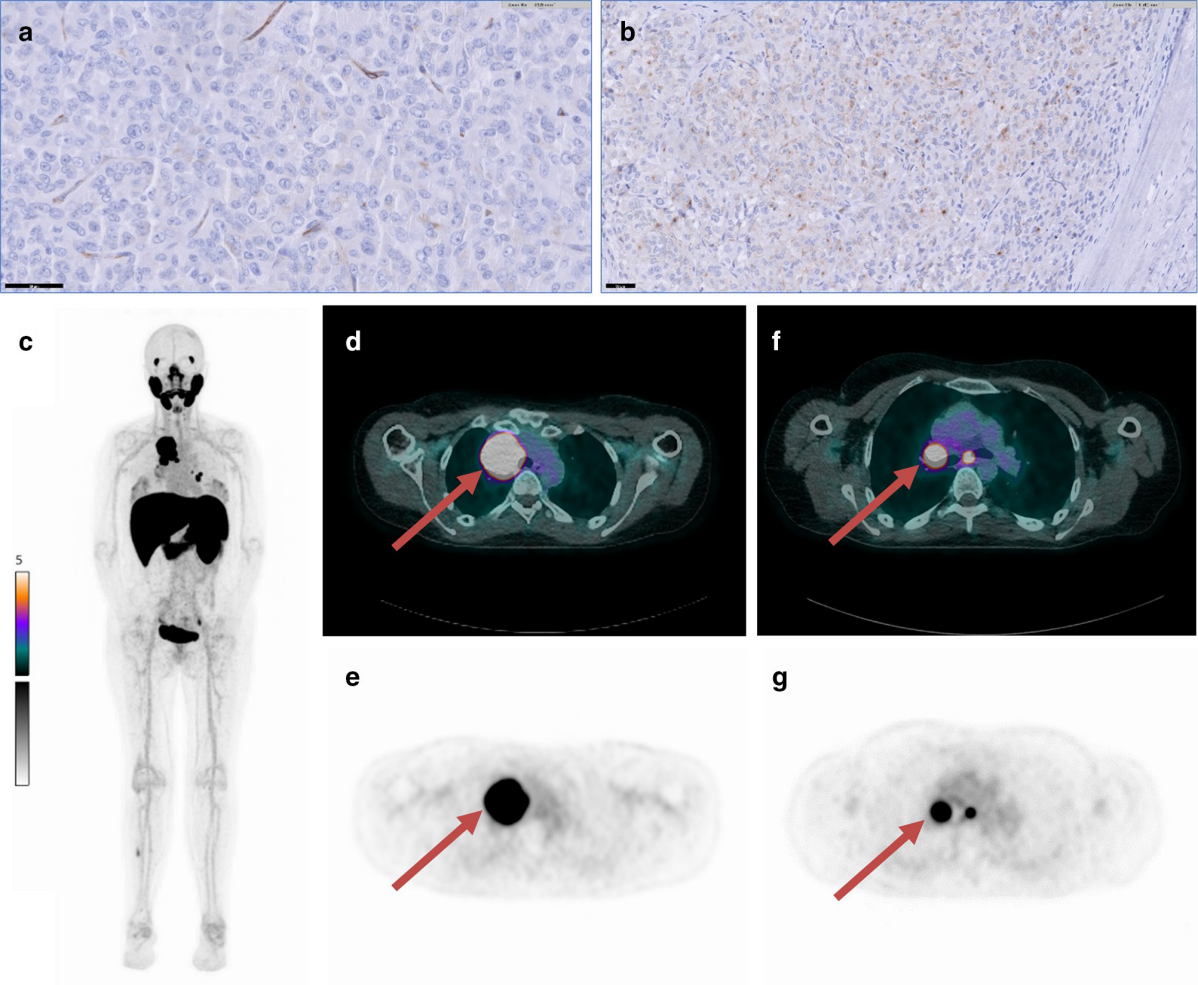
A 33-year-old female with an epithelioid malignant peripheral nerve sheath tumour in the right ankle. PSMA immunohistochemistry on the biopsy material of this tumour revealed both focal neovascular PSMA expression (**A**) and cytoplasmic, dot-like PSMA staining in 30% of tumour cells (**B**).Nineteen months post-operatively, conventional imaging detected multiple mediastinal and pulmonary metastases. A [^18^F]JK-PSMA-7 PET/CT scan was experimentally made to assess tumoural PSMA-tracer binding. The [^18^F]JK-PSMA-7 PET/CT scan showed good tracer accumulation in mediastinal metastases (SUV_max_ = 21.5, see D and E), pulmonary metastases (SUV_max_ = 14.8, see F and G) and one bone metastasis in the right fibula (SUV_max_ = 3.0). C: whole-body maximal intensity projection, D and F: fused PET/CT images, E and G: PET images. These results from the Leiden University Medical Centre (Leiden, The Netherlands) have not been published before. Informed consent for publishing the data and images was obtained from the patient.

First of all, three liposarcomas have shown PSMA uptake; a well-differentiated, dedifferentiated and pleomorphic liposarcoma.^31–33^ It is remarkable that in the dedifferentiated liposarcoma, PSMA seemed to differentiate between the lipomatous and non-lipomatous regions, where the latter was PSMA-positive. The highest tumour-to-background ratio was seen on the PSMA PET/CT scan of pleomorphic liposarcoma (SUV_max_ = 13). Plouznikoff et al. and Mathew et al. reported two patients with PSMA-positive undifferentiated pleomorphic sarcoma in the left obturator muscle and the right posterior chest wall, respectively, one of which was likely radiation-induced.^34,35^ As for bone sarcomas, one Ewing sarcoma and two osteosarcomas with PSMA uptake have been described.^36–38^ Two of them were females, for which the PSMA PET/CT scans were specifically made to assess the amount of PSMA-tracer binding in the tumour, while in all other case reports the PSMA-avid sarcomas were incidental findings in patients with prostate cancer. In one patient with osteosarcoma, PSMA could differentiate between areas of fibrous dysplasia and areas of malignant transformation to osteosarcoma in the skull bones. When looking at sarcomas in a metastatic setting, one of the osteosarcoma patients and additionally one patient with angiosarcoma and two patients with leiomyosarcoma had multiple PSMA-avid metastases.^39–41^ Interestingly, especially in these patient cases high PSMA uptake with high SUV_max_ values were reported. The highest SUV_max_ values were described in high-grade osteosarcoma, localized in the sternum and multiple lung, bone and liver metastases (SUV_max_ values of 10.6–35.1). An example of one of our own patients with PSMA-avid metastatic sarcoma is shown in Figure 4.

The potential pitfall of PSMA-targeted PET/CT imaging is that not all PSMA-tracer uptake besides physiological uptake can be attributed to malignant lesions. Occasionally, PSMA expression and PSMA-tracer uptake are seen in benign neoplasms, such as haemangiomas and schwannomas.^22,27^ Without careful interpretation, such lesions might be easily mistaken for metastases.^42–46^ It is important to take this possibility into account while interpreting PSMA PET/CT scans of sarcoma patients, as this would otherwise lead to false-positive findings.

### PSMA-targeted therapy in sarcomas

While for prostate cancer PSMA-RLT is still often given in a research setting, already two case reports have been published describing the administration of ^177^Lu-PSMA-RLT to a patient with metastasized leiomyosarcoma, both also described in Table 2.^40,41^

**Figure 5.**
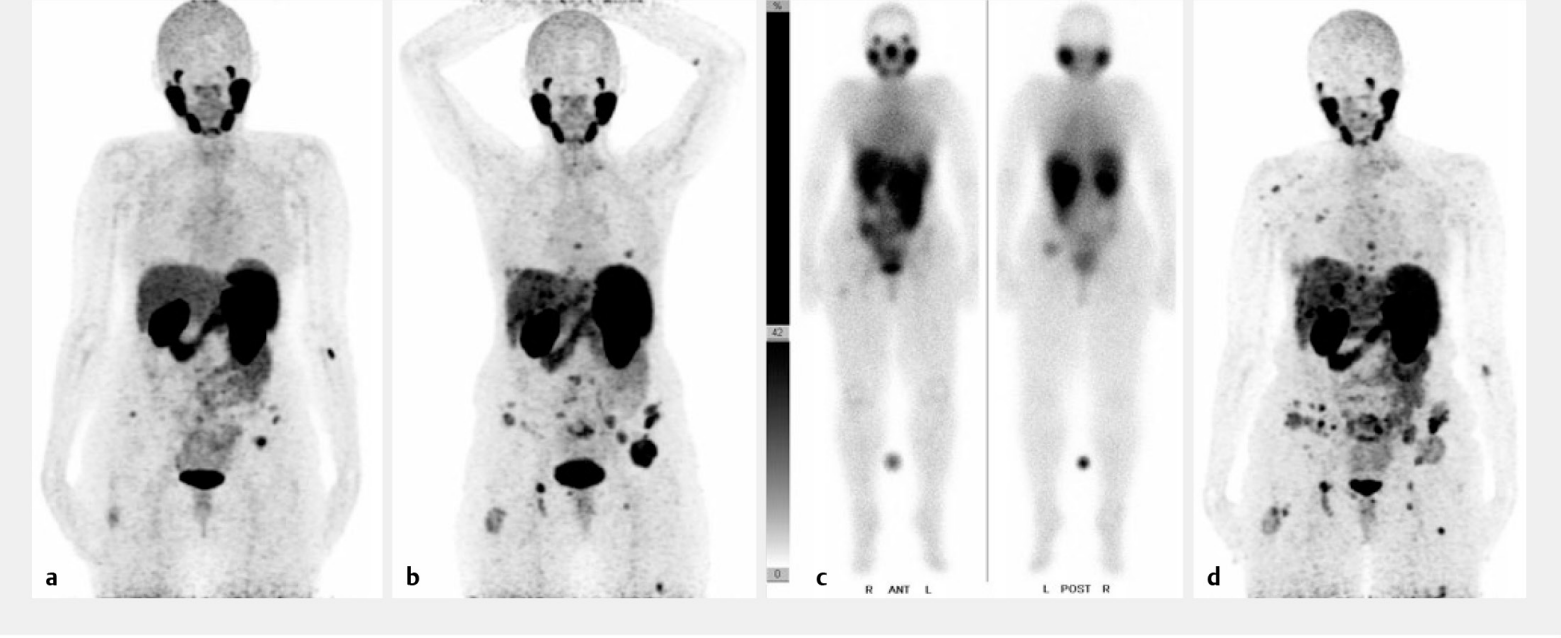
A 50-year-old female patient with metastasized leiomyosarcoma has received one application of 6.0 GBq [^177^Lu]Lu-PSMA-617. This figure was obtained from Jüptner et al.^40^ **a** First 68Ga-PSMA-PET/CT scan in July 2017. First 68Ga-PSMA-PET/CT scan of the patient with several lung and bone metastases andsingular lesions in the liver, in the left gluteus maximus muscle and in the right vastus lateralis muscle. **b** Second 68Ga-PSMA-PET/CT scan in April2018. Progression of the disease with increasing PSMA accumulation in the existing metastases and multiple newly emerged lesions. **c** Intratherapeuticwhole body scan 24 hours after injection of 6 GBq 177Lu-PSMA-617. Poor fixation of the radiotracer in the multiple metastases. A dosimetricalphantom is placed between the legs. R = right, L = left, ANT = anterior, POST = posterior. **d** Posttherapeutic 68Ga-PSMA-PET/CT scan in August2018. Progress of the PSMA-positive lesions with global proceeding lung, liver, bone and muscle metastases. The leading lesion in the left gluteusmaximus muscle decreased because of previous external beam radiation. Copyrights for this figure were acquired through the Copyright Clearance Centre.

The first patient was a 50-year-old female who was diagnosed with a leiomyosarcoma of the vena cava inferior in 2015 (TNM pT2pN0(0/9)pR0 FNCLCC Grade 1).^40^ In 2016, the patient underwent selective internal radiotherapy for several liver metastases. In 2017, routine diagnostics revealed new metastatic lesions in bone, lung and muscle. As routine systemic therapies were denied by the patient, a diagnostic PSMA PET/CT scan was performed to assess the feasibility of PSMA-RLT. Because of low SUV_max_ values, re-evaluation with PSMA PET/CT was done one year later, which showed clear progression of disease with multiple new lesions and increased SUV_max_ values. The lesions in both lungs, liver, left gluteus maximus muscle, right vastus lateralis muscle and multiple bone lesions were treated with one application of 6.0 GBq [^177^Lu]Lu-PSMA-617. The radionuclide therapy was well tolerated by the patient, but intratherapeutic whole body scans revealed moderate PSMA uptake in the left gluteus maximus muscle and left os ilium and weak uptake in all other lesions. Therefore, no other [^177^Lu]Lu-PSMA-617 cycles were given. Three months later, further progression of the disease was seen. The images of this patient are shown in Figure 5.

Another female patient was diagnosed with uterine leiomyosarcoma with peritoneal metastases in 2018, in her mid-50s.^41^ After several palliative chemotherapy treatments, tumour progression was observed. Due to the presence of tumour-infiltrating lymphocytes and a lack of standard treatment options, off-label nivolumab treatment was initiated in February 2021. Four months later, the tumour growth rate was +36.5% per month (compared to +23.8% per month before nivolumab was started). To test the feasibility of PSMA-RLT, a PSMA PET/CT scan was performed, which showed PSMA uptake in lung, adrenal and peritoneal lesions. Two cycles of [^177^Lu]Lu-PSMA-I&T with a two-month interval were administered in combination with nivolumab. Four months after the last dose, the tumour growth rate reduced to +11.3% per month. The lung lesion with the highest PSMA uptake (SUV_max_ = 8.9) showed a considerable size reduction. At this time, nivolumab treatment was stopped. The administered dose of [^177^Lu]Lu-PSMA-I&T and its tumour retention time, however, were not described in this case report.

### Future perspectives

To assess the feasibility of PSMA-targeted imaging and therapy in patients with sarcomas, it is important to know whether or not PSMA expression is seen in the tumour, and which factors determine effective PSMA-RLT. Previously published literature reveals that not all sarcomas, but definitely a subgroup of sarcomas show (strong) PSMA expression in their tumour-associated neovasculature, and (high) PSMA-tracer uptake on PET/CT imaging. These results provide a rationale that PSMA-RLT can be successful in a selection of sarcoma patients.

In prostatic tissue, PSMA expression is upregulated in case of malignant transformation and is further upregulated in more advanced stages of disease. Several studies show that PSMA expression is an independent prognostic factor in prostate cancer patients, associated with worse survival.^16^ Similar patterns can be observed in sarcomas. PSMA immunohistochemistry studies revealed that sarcoma types had significantly higher PSMA expression compared to tumour types with benign or intermediate biological potential.^27^ Furthermore, in osteosarcomas, PSMA expression was significantly associated with tumour size, the presence of pulmonary metastasis and a worse 5 year survival rate.^28^ In previously published case reports of PSMA-tracer uptake in sarcomas on PET/CT imaging, PSMA was able to differentiate between dedifferentiated liposarcoma and lipomatous regions^32^ and between osteosarcoma and fibrous dysplasia,^37^ showing visibly higher PSMA-tracer uptake in the malignant lesions. Although not every case report has described SUV_max_ values, metastasized sarcomas seem to have more intense PSMA-tracer uptake compared to non-metastasized sarcomas.^38,40^ Lastly, one patient with metastasized leiomyosarcoma who received multiple PSMA PET/CT scans showed a noticeable increase in SUV_max_ values after one year of disease progression.^40^ These observations suggest that if present, PSMA expression seems to be highest in patients with aggressive and/or advanced sarcomas, similar to prostate cancer. These are also the patient groups that are in highest need of new therapeutic options and could benefit the most from PSMA-RLT.

Currently, there is no evidence for an optimal selection method yet to determine whether a sarcoma patient is likely to respond to PSMA-RLT. The most evident option seems to select eligible patients by using PSMA immunohistochemistry on biopsy or resection material to identify tumours with high PSMA expression. In prostate cancer, the percentage of PSMA-expressing tumour cells correlates significantly with SUV_max_ on PSMA PET/CT imaging.^47^ However, no studies have investigated the correlation between PSMA immunohistochemistry and SUV_max_ on PSMA PET/CT imaging in sarcomas yet. Therefore, no definition of ‘high PSMA expression’, enough for significant PSMA-tracer uptake in sarcomas on PSMA PET/CT imaging, is available. As sarcomas have mainly shown PSMA expression on the neovascular endothelial cells instead of on the tumour cells itself (with some rare exceptions), the radiotracer uptake is expected to be less than in prostate carcinoma. However, the case reports of *e.g.* Militano et al., Can et al. and Jüptner et al. show that high SUV_max_ values over 12 can be reached in sarcomas, which generally is considered enough to explore the possibility of PSMA-RLT.^48,49^ Additionally, several case reports that describe ^177^Lu-PSMA-RLT treatment in other non-prostatic malignancies with neovascular PSMA expression, such as glioblastoma multiforma, show that good tumour retention (in a lesion with a SUV_max_ of 10.3) and a significant reduction in tumour size and symptoms can be achieved, with acceptable toxicity.^50,51^ It is also important to consider that a direct correlation between PSMA-tracer uptake on PSMA PET/CT imaging and the intratumoural dose after PSMA-RLT is not self-evident, as the 6.0 GBq of [^177^Lu]Lu-PSMA-617 that was administered to a patient with metastasized leiomyosarcoma, showed limited retention in the tumour 24 h after injection compared to the diagnostic ^68^Ga-PSMA PET/CT scan that was obtained beforehand.

Although the currently available literature suggests that there might be a selection of sarcoma patients that could benefit from PSMA-RLT, it should be realized that this suggestion is based on a limited amount of patient studies and a few number of clinical cases. For a successful treatment with PSMA-RLT, two factors are of great importance: 1) the achieved intratumoural radioactive dose, which is influenced by multiple factors such as the total administered radiation dose, the tumoural reachability, the radiotracer’s affinity towards PSMA, its retention time and the level of PSMA expression, and 2) the radiosensitivity of the tumour. Further investigation is necessary to gain knowledge about each of these aspects regarding sarcoma patients, *e.g.* by comparing different PSMA-targeting antibodies and tracers, evaluating their tumour dosimetry and assessing PSMA-RLT efficacy and toxicity profiles with different doses. However, first of all, more insight is needed into what tumour characteristics predict significant intratumoural binding of PSMA-ligands. For this, currently, one trial is recruiting patients to compare PSMA immunohistochemistry in biopsy material with, in case of high PSMA expression, PSMA-tracer accumulation on PET/CT imaging (clinicaltrials.gov, NCT05522257). Furthermore, in end-stage metastatic prostate cancer, ^177^Lu- and ^225^Ac-labelled PSMA have proven to prolong overall and progression-free survival with acceptable toxicity.^20,21^ Recently, the first results on ^89^Zr-labelled PSMA ligands in prostate cancer have been published, having the advantage of a longer half-life and allowing acquisition at later time points after injection.^52,53^ This might result in higher tumour-to-background ratios, better assessment of tumour retention and improved patient selection for PSMA-RLT compared to ^18^F or ^68^Ga. Developments in this field and the increasing knowledge about the role of PSMA in different neoplasms should be closely monitored and used for the rationale regarding PSMA-RLT in sarcoma patients as well.

## Conclusion

Strong PSMA expression and good PSMA-tracer accumulation are observed in a selection of sarcoma patients, which seems to be more prevalent in aggressive and advanced sarcomas. These patient groups are also in highest need of new therapeutic options, as their 5-year survival rates are low. The results from previous literature have laid the foundation for further research that is needed to investigate the possible efficacy of PSMA-RLT in sarcomas and how eligible sarcoma patients could be selected in a reliable way. Hopefully, additional insights will be given by the currently recruiting prospective study (clinicaltrials.gov, NCT05522257).
